# Downregulated circular RNA hsa_circ_0005797 inhibits endometrial cancer by modulating microRNA-298/Catenin delta 1 signaling

**DOI:** 10.1080/21655979.2021.2013113

**Published:** 2022-02-11

**Authors:** Yating Liu, Hongying Yuan, Tao He

**Affiliations:** a Department of Gynecology, Luoyang Maternal and Child Health Hospital, Luoyang, Henan, China; b Department of Gynecology, The First Affiliated Hospital, and College of Clinical Medicine of Henan University of Science and Technology, Luoyang, China; c Medicine College, Henan University of Science and Technology, Luoyang, China; d Department of Gynecology New District Hospital,The First Affiliated Hospital, and College of Clinical Medicine of Henan University of Science and Technology, Luoyang, China

**Keywords:** Endometrial carcinoma, hsa_circ_0005797, miR-298, CTNND1

## Abstract

Abnormal expression of circular RNA (circRNA) expression has been implicated in endometrial cancer (EC) progression. Thus, investigation of the mechanism of hsa_circ_0005797 during EC etiology may provide new insight into the treatment of EC. In the present study, we found that hsa_circ_0005797 expression was significantly increased in EC biological samples and cell lines, whereas its downregulation inhibited *in vitro* tumor cells proliferation and invasion phenotypes and suppressed tumor formation in nude mice. In mechanism, we characterized hsa_circ_0005797 as an miR-298 sponge, with CTNND1 identified as a target of miR-298. Our rescue assay data further revealed that hsa_circ_0005797 silencing inhibited EC cells proliferation and invasion via miR-298/CTNND1 signaling. In conclusion, our study confirmed hsa_circ_0005797 is a poor prognostic factor for EC and modulates EC phenotypes by regulating the hsa_circ_000579/miR-298/CTNND1 signaling, which provides potential treatment targets for EC

## Introduction

Globally, endometrial cancer (EC) is a highly prevalent cancer in the female reproductive tract, and the second major cause of gynecological cancer-related mortality [1,2]. Despite considerable advancements in therapeutic EC strategies, prognoses for patients with EC with an advanced tumor grade or metastasis remain unsatisfactory [3,4]. Therefore, a comprehensive understanding of underlying molecular mechanisms implicated in tumor progression will provide new therapeutic opportunities for EC tumor diagnostics and treatment.

Circular RNAs (circRNAs) are endogenous non-coding RNAs with closed circular structures [5,6]. Increasing evidence has suggested circRNAs aberrant expression exert critical roles during tumorigenesis [7,8]. For example, circRNA-002178 functioned as a competitive endogenous RNA (ceRNA) to promote PDL1/PD1 levels during lung cancer progression [9]. Similarly, circPRRC2A promoted angiogenesis and metastasis via epithelial-mesenchymal transition (EMT) to increase TRPM3 levels in renal cancer [10]. Also, circSLC8A1 acted as a miR-130b/miR-494 sponge to suppress bladder cancer advancement by modulating PTEN [11]. These studies suggested that circRNAs play important roles in tumorigenesis. Hence, the roles and underlying mechanisms of hsa_circ_0005797 in EC remain unclear.

Catenin delta 1 (CTNND1) is a key regulator of cell-cell adhesion that associates with and regulates the cell adhesion properties of both C-, E- and N-cadherins [12]. Recently, increasing studies reported that CTNND1 functioned as an oncogene during cancer occurrence and development [12,13]. For example, CTNND1 overexpression in primary liver cancer (hepatocellular carcinoma; HCC) promoted cancer phenotypes via Wnt/β-catenin axis activation [14]. Also, miR-425-5p stimulated tumor growth and metastasis via CTNND1-mediated β-catenin signaling activation and EMT mechanisms in colorectal cancer [13]. Similarly, CircMAST1 promoted key HCC phenotypes by sponging miR-1299 and regulating CTNND1 levels [15]. These studies implicated CTNND1 as an important molecular player during tumor progression.

In this work, hsa_circ_0005797 was highly elevated in EC samples. By sponging miR-298, hsa_circ_0005797 modulated CTNND1 expression, and regulated EC cells proliferation, invasion in vitro, and tumor growth in vivo. Thus, we identified hsa_circ_0005797/miR-298/CTNND1 as a key signaling axis facilitating aggressive EC growth, providing a novel therapeutic strategy in EC treatment

## Materials and Methods

### Tissues samples

In total, 48 samples (35 EC samples and 13 normal endometrial samples) were used in this study. EC samples were gathered from patients undergoing surgery at the First Affiliated Hospital, and College of Clinical Medicine of Henan University of Science and Technology and Luoyang Maternal and Child Health Hospital. All patients had given their written informed consents. None of the patients had received chemotherapy or radiotherapy prior to surgery. All producers were approved by the human Ethics Committee of our hospital. The detailed clinicopathological features are described in Table 1.

**Table 1.** Clinical features of endometrial cancer patients

Factors		Numbers
Age (years)		26 9
	≥ 50	
	< 50	
Tumor size (cm)		23 12
	≥ 4 cm	
	< 4 cm	
FIGO stage		15 20
	I–II	
	III–IV	
Lymph node metastasis		15 20
	Positive	
	Negative	

### Cell culture and transfection

HEC-1-B, AN3-CA, KLE, HEC1-A, and Ishikawa EC cell lines, and human endometrial endothelial cells (HEEC) were purchased from the American Type Culture Collection (ATCC). Cells were grown in Dulbecco’s Modified Eagle Medium (DMEM, Thermo Fisher Scientific, MA, USA) plus 10% fetal bovine serum (FBS, Thermo Fisher Scientific) at 37°C in 5% CO_2_.

GenePharma (Shanghai, China) provided small interfering RNA (siRNA) hsa_circ_0005797 (si-circ_0005797#1:5’-GCTTGTTTGAGGTTGCACGTT-3’; si-circ_0005797#2: 5’-GTTTGAGGTTGCACGTTTTCA-3’; si-circ_0005797#1:5’- TTGAGGTTGCACGTTTTCAAA-3’), si-negative control (si-NC), miR-298 mimics (cat no.: 219,600; Qiagen), miR-298 inhibitors (cat no.: 219,300; Qiagen), and corresponding negative controls (cat no.: 1,027,271). Lipofectamine 3000 was used for transfections (Invitrogen, Carlsbad, CA, USA).

### RNase R and actinomycin D assays

RNase R studies were conducted using RNase R (Epicenter, Madison, WI, USA) for 20 min. EC cells were treated with actinomycin D (Sigma-Aldrich, St. Louis, MO, USA) for 1 day. After treatments, hsa_circ_0005797 and ATP2C1 levels were detected.

### Quantitative real-time polymerase chain reaction (qRT-PCR)

Total RNA was extracted using TRIzol (Invitrogen, Waltham, MA, USA), concentrations determined, and reversely transcribed to cDNA using a Prime Script RT Kit (Takara, Dalian, China). The miRcute Plus miRNA first strand cDNA synthesis kit (TIANGEN, Beijing, China) was used to generate miRNA cDNA. Using cDNA as the template, detection was performed using a RT fluorescence quantitative PCR kit. Data were processed using 2^−ΔΔCt^ calculations. U6 was used as an endogenous control for miR-298, and GAPDH was the endogenous control for hsa_circ_0005797 and CTNND1. The primer sequences were shown as hsa_circ_0005797, F, 5’- AGCATACACTTGCCCGAGAC-3’; R, 5’- GGAATCATTGTCTCATTTTCACC-3’. miR-298, F, 5’- TCAGGTCTTCAGCAGAAGC-3’; R, 5’- TAGTTCCTCACAGTCAAGGA-3’. CTNND1, F, 5’- ATGTTTGCGAGGAAGCCGC-3’; R, 5’- CGAGTGGTCCCATCATCTG-3’. GAPDH, F, 5’-GACAGTCAGCCGCATCTTCT-3’; R, 5’-GCGCCCAATACGACCAAATC-3’. U6, F, 5’- ATACAGAGAAAGTTAGCACGG-3’; R, 5’- GGAATGCTTCAAAGAGTTGTG-3’.

### Western blotting

RIPA lysis buffer (Beyotime, Shanghai, China) was used to extract protein. Then, 50 μg was separated by 10% sodium dodecyl sulfate-polyacrylamide electrophoresis (SDS-PAGE), transferred to polyvinylidene fluoride (PVDF) membranes which were probed overnight at 4°C with primary antibodies: CTNND1 (1:500, Abcam, ab92514) and GAPDH (1:3000, Abcam, ab8245). Membranes were then probed for 2 h at room temperature with a horse radish peroxidase conjugated secondary antibody. Protein signals were detected using an enhanced chemiluminescent detection kit (Pierce Biotechnology, Rockford, IL, USA).

### Cell viability assays

Cell proliferation was determined by Cell Counting Kit-8 (CCK-8; Dojindo, Kumamoto, Japan) assay. Briefly, 5000 transfected EC cells/well in 96-well plates. Then, 10 μL CCK-8 solution was added to each well and the plate incubated for 2 h at 37°C in 5% CO_2_. Absorbance at 450 nm was measured on a microplate reader (Bio-Rad, Hercules, CA, USA).

Colony formation ability was determined by a colony formation assay. In brief, transfected EC cells in 6-well plates (1000 cells/well) were grown for 2 weeks. After formaldehyde fixation, colonies were stained in 0.5% crystal violet for counting.

### Cell invasion assay

This phenotype was investigated using 24-well Transwell plates (8 μm; BD Biosciences, San Jose, CA, USA). Briefly, EC cells in serum-free DMEM were added to a Transwell upper chamber (coated with 1 mg/mL matrigel, Corning, NY, USA). After 24 h incubation, invading cells were fixed, then stained in 0.1% crystal violet. Five random fields in each Transwell were selected for counting and statistical analysis.

### Dual-luciferase reporter assay

Wild type (WT) and/or mutant (MUT) hsa_circ_0005797 or CTNND1 3’ untranslated region (UTR) fragments containing putative miR-298 binding sites were generated and inserted into pmirGLO dual-luciferase reporter plasmids. For the reporter assay, EC cells were co-transfected with reporter plasmids, miR-298 mimics and/or miR-NC reagents. After two days growth, lysed cells underwent relative luciferase activity assay (Promega, Madison, WI, USA).

### RNA pulldown assay

Biotin-labeled hsa_circ_0005797 probe and control probe (Sangon Biotech, China) were used for circRNA pull-down and the assay was performed as mentioned previously [16].

### RNA immunoprecipitation (RIP)

According to the manufacturer’s instructions and a previous study, the Magna RIPTM RNA-Binding Protein Immunoprecipitation Kit (Millipore, Temecula, CA, USA) was used for RIP assays [17]. Immunoprecipitated RNAs were quantified to assess hsa_circ_0005797 and miR-298 expression levels.

### Xenograft mouse model

4 weeks old female BALB/c-nu mice (Shanghai Lab Animal Research Center, Shanghai, China) were used to assess tumor growth. Animal studies were ethically sanctioned by our hospital. Briefly, 5 × 10^6^ transfected EC cells were subcutaneously injected into a right lower mouse limb. Tumor sizes were measured every seven days; tumor volume (mm^3^) = (length x width^2^)/2. After 42 days, tumors were removed from anesthetized mice and tumor weights were recorded.

### Statistical analyses

All analyses were conducted using SPSS ver. 19.0 software (IBM, Armonk, NY, USA). Data were represented as the mean ± standard deviation (SD). Student’s t tests were used to compare significant differences between two groups, whereas multiple group data were tested using two-way ANOVA. A P < 0.05 value was accepted as statistically significant.

## Results

### Hsa_circ_0005797 levels are elevated in EC

**Figure 1.**
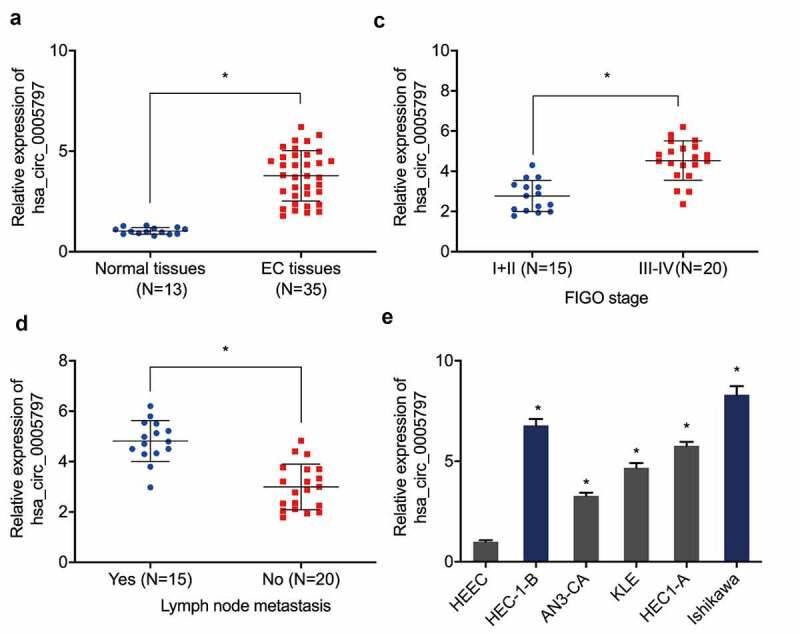
**Hsa_circ_0005797 is elevated in patients with EC**. (a) Hsa_circ_0005797 expression in EC tissue. (b, c) Elevated hsa_circ_0005797 levels in EC tissues are associated with advanced Federation of Gynecology and Obstetrics (FIGO) staging, and lymph node metastasis. (d) Hsa_circ_0005797 expression in EC cells. *P < 0.05.

Previous research reported that hsa_circ_0005797 was elevated in EC tissues [18], however, its specific functions and mechanisms in this cancer are still unclear. Our expression analyses indicated hsa_circ_0005797 levels in EC tissue were higher than normal endometrium specimens (Figure 1(a)). Elevated hsa_circ_0005797 levels were related to advanced Federation of Gynecology and Obstetrics (FIGO) staging, and lymph-node metastasis in patients with EC (Figures 1(b,c)). Similarly, hsa_circ_0005797 levels in all 5 EC cell lines were higher than HEEC cells (Figure 1(d)).

**Figure 2.**
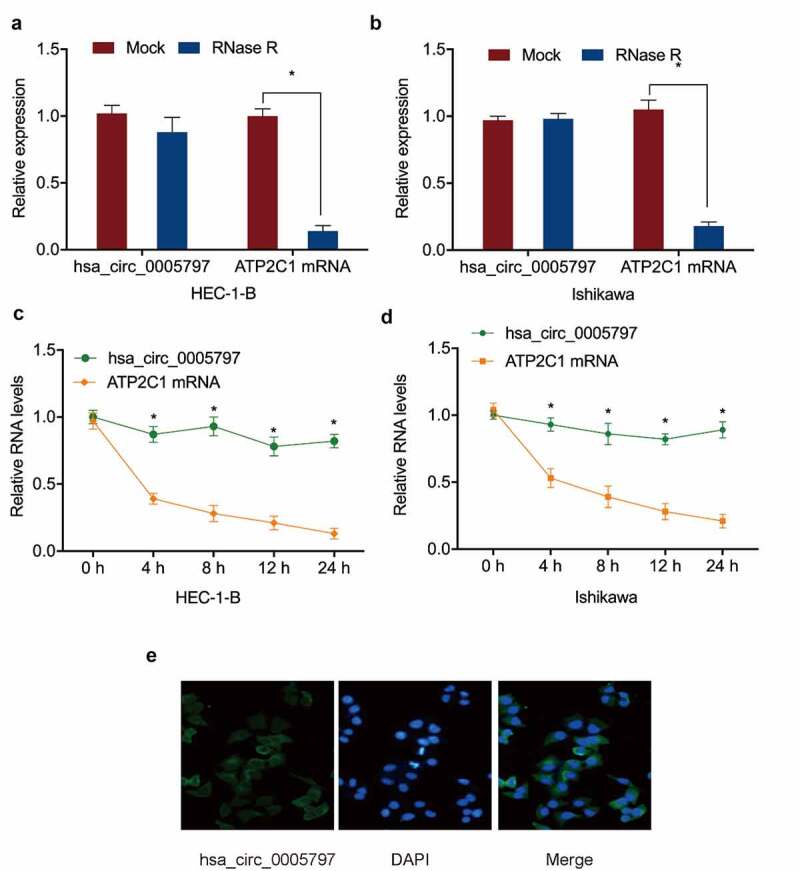
**Hsa_circ_0005797 characteristics**. (a, b) Hsa_circ_0005797 and ATP2C1 in EC cells treated with RNase R. (c, d) Hsa_circ_0005797 and ATP2C1 levels in EC cells treated with Actinomycin D. (e) Hsa_circ_0005797 localization to the cytoplasm in EC cells. *P < 0.05.

To confirm hsa_circ_0005797 was a circRNA, RNase R digestion and actinomycin D assays were performed. Linear ATP2C1 RNA levels in the RNase R-treated group were markedly lower than the mock group, whereas hsa_circ_0005797 were unaffected by RNase R treatment (Figures 2(a,b)). The actinomycin D assay further confirmed these observations (Figure 2(c,d)). Next, we revealed hsa_circ_0005797 was localized to the cytoplasm in EC cells (Figure 2(e)). Thus, the above findings indicated that the upregulation of hsa_circ_0005797 may exert an oncogenic effect on EC progression.

### Hsa_circ_0005797 promotes EC cells proliferation and invasion

**Figure 3.**
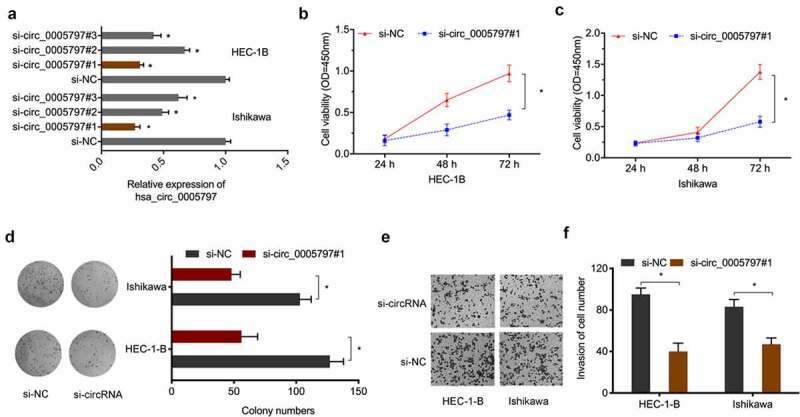
**The impact of hsa_circ_0005797 on EC growth and invasion**. (a) EC cells transfected with si-NC or si-circ_0005797. (b-d) EC cells viability and colony formation. (e, f) EC cells invasion. *P < 0.05.

To determine the oncogenic function of hsa_circ_0005797 in EC, si-circ_0005797 was transfected into HEC-1B and Ishikawa cells (Figure 3(a)). From CCK-8 and colony formation studies, hsa_circ_0005797 silencing significantly inhibited *in vitro* EC cells proliferation abilities (Figure 3(b-d)). Also, hsa_circ_0005797 silencing markedly reduced EC cells invasion capacities when compared with control group (Figure 3(e,f)). Therefore, these results demonstrated that hsa_circ_0005797 silencing efficiently inhibited EC cells progression.

### Hsa_circ_0005797 is a sponge for miR-298

**Figure 4.**
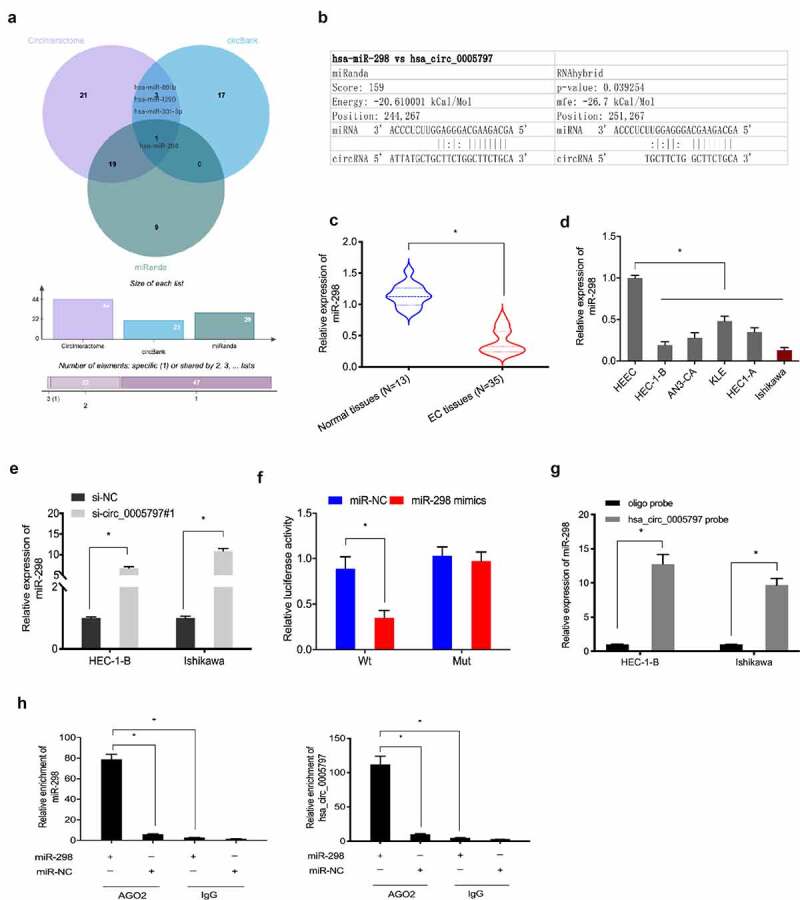
**MiR-298 is a target of hsa_circ_0005797**. (a) Venn diagram of potential targets of hsa_circ_0005797. (b) Binding sites between hsa_circ_0005797 and miR-298. (c, d) MiR-298 expression levels were decreased in EC tissues and cell lines. (e) Has_circ_0005797 suppression increased miR-298 expression in EC cells. (f) miR-298 mimics reduced the relative luciferase activity of hsa_circ_0005797-WT in EC cells. (g, h) The relationship between hsa_circ_0005797 and miR-298 was confirmed by RNA pull-down and RIP assays. *P < 0.05.

To explore hsa_circ_0005797 mechanisms during EC progression, circBank, CircInteractome and miRanda databases were explored. We observed that miR-298 could be a potential target miRNA of hsa_circ_0005797 (Figure 4(a,b)). Additionally, miR-298 levels in EC samples and cell lines were significantly downregulated when compared with normal tissues and HEEC cells (Figure 4(c,d)). Expression analyses indicated that hsa_circ_0005797 silencing significantly elevated miR-298 levels in HEC-1B and Ishikawa cells (Figure 4(e)). Luciferase reporter assay revealed that miR-298 overexpression significantly reduced the relative luciferase activity of hsa_circ_0005797-WT plasmid in EC cells (figure 4(f)). Moreover, RNA pull-down and RIP assays confirmed the relationship between hsa_circ_0005797 and miR-298 in EC cells (Figure 4(g,h)). Thus, these observations indicated hsa_circ_0005797 might function as a sponge for miR-298 in EC progression.

### CTNND1 is a target of miR-298

**Figure 5.**
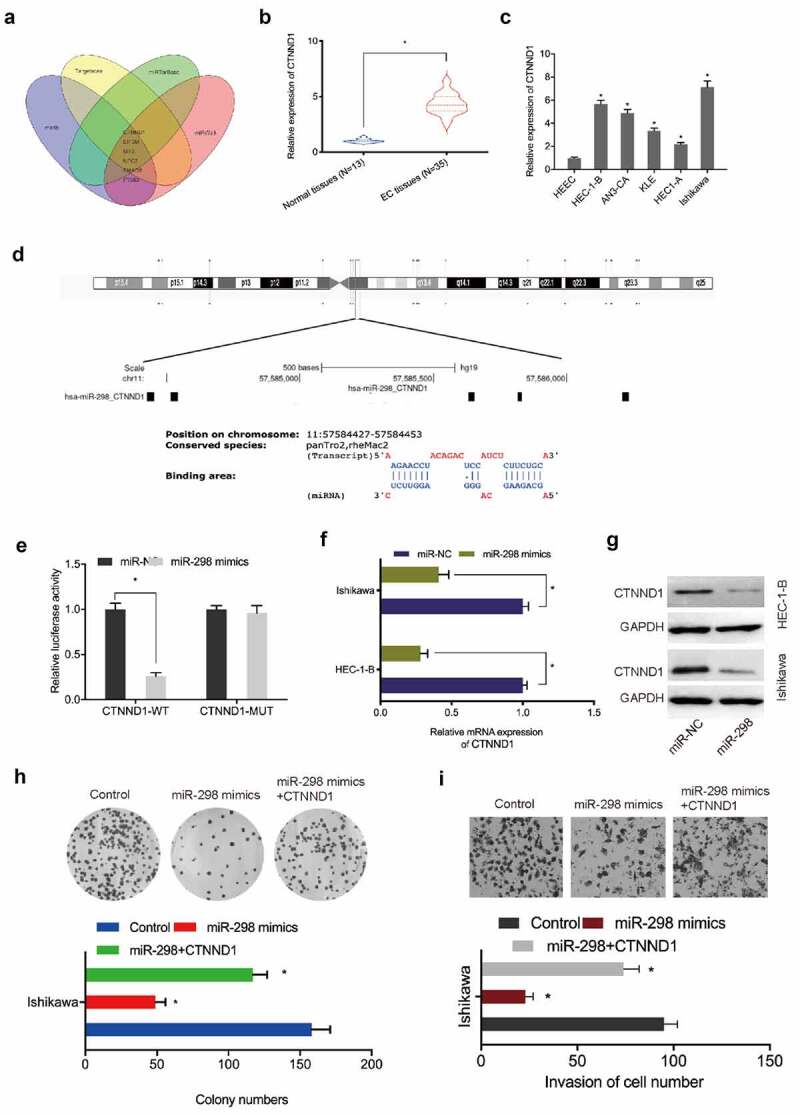
**CTNND1 is a target of miR-298**. (a) Venn diagram of potential targets of miR-298. (b, c) CTNND1 expression in EC tissues and cell lines. (d) Binding sites between CTNND1 and miR-298. (e) The targeted relationship between CTNND1 and miR-298 was confirmed by Dual-luciferase reporter assay. (f, g) CTNND1 expression levels in Ishikawa cells transfected with miR-NC or miR-298 mimics. (h, i) Overexpressed CTNND1 abolished miR-298 mimics functions toward key EC phenotypes. *P < 0.05.

To identify putative miR-298 targets, we used Targetscan7, mirTarBase, and miRWalk, and mirdb databases to identify 6 potential target genes, with CTNND1 chosen for further study (Figure 5(a-d)). Expression analyses indicated that CTNND1 levels in EC tissues and cell lines were significantly elevated (Figure 5(b,c)). Luciferase reporter assays revealed that miR-298 overexpression reduced relative luciferase activity in CTNND1-WT cells (Figure 5(e)). Additionally, miR-298 overexpression reduced CTNND1 mRNA and protein levels in EC cells (figure 5(f,g)). Moreover, functional assays showed that miR-298 overexpression suppressed Ishikawa cells proliferation and invasion abilities, but these were abolished by elevating CTNND1 overexpression (Figure 5(h,i)). These observations suggested CTNND1 was a direct target of miR-298 in EC progression.

### Hsa_circ_0005797/miR-298/CTNND1 signaling in EC

**Figure 6.**
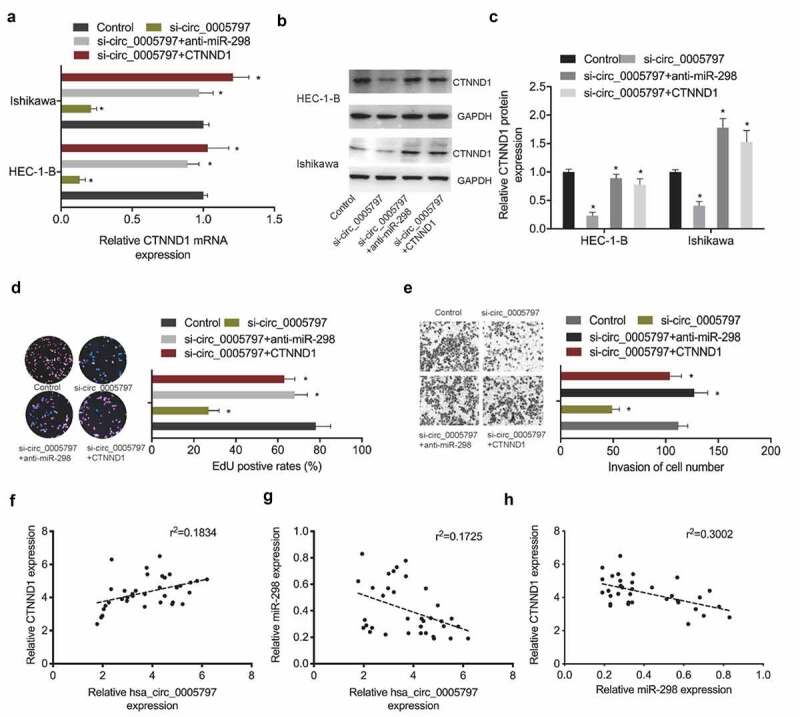
**Hsa_circ_0005797/miR-298/CTNND1 signaling in EC**. (a-c) CTNND1 levels in EC cells transfected with si-NC, si-circ_0005797, si-circ_0005797 + miR-298 inhibitors (anti-miR-298) or si-circ_0005797 + CTNND1. (d, e) MiR-298 inhibitors or CTNND1 overexpression abolished hsa_circ_0005797 suppression effects on Ishikawa cells proliferation and invasion. (f-h) The correlation among hsa_circ_0005797, miR-298, and CTNND1 in EC tissues. *P < 0.05.

To verify hsa_circ_0005797/miR-298/CTNND1 signaling in EC progression, several key assays were conducted. qRT-PCR and Western blot assays revealed that CTNND1 levels were greatly reduced in EC cells in the absence of hsa_circ_0005797, while suppression of miR-298 or CTNND1 overexpression partially reversed these effects (Figure 6a-6c). Next, functional assays revealed that hsa_circ_0005797 reduced Ishikawa cells proliferation and invasion abilites, while miR-298 inhibitors or CTNND1 overexpression abolished hsa_circ_0005797 suppression effects in EC cells (Figure 6(d,e)). Furthermore, correlation analysis showed that hsa_circ_0005797 was positively associated with CTNND1 expression and negatively associated with miR-298 expression in EC tissues, as well as CTNND1 expression was negatively associated with miR-298 expression in EC tissues (figure 6f-6h). Combined, hsa_circ_0005797 putatively promoted EC progression via miR-298/CTNND1 signaling.

### *Hsa_circ_0005797 silencing inhibits tumor growth* in vivo

**Figure 7.**
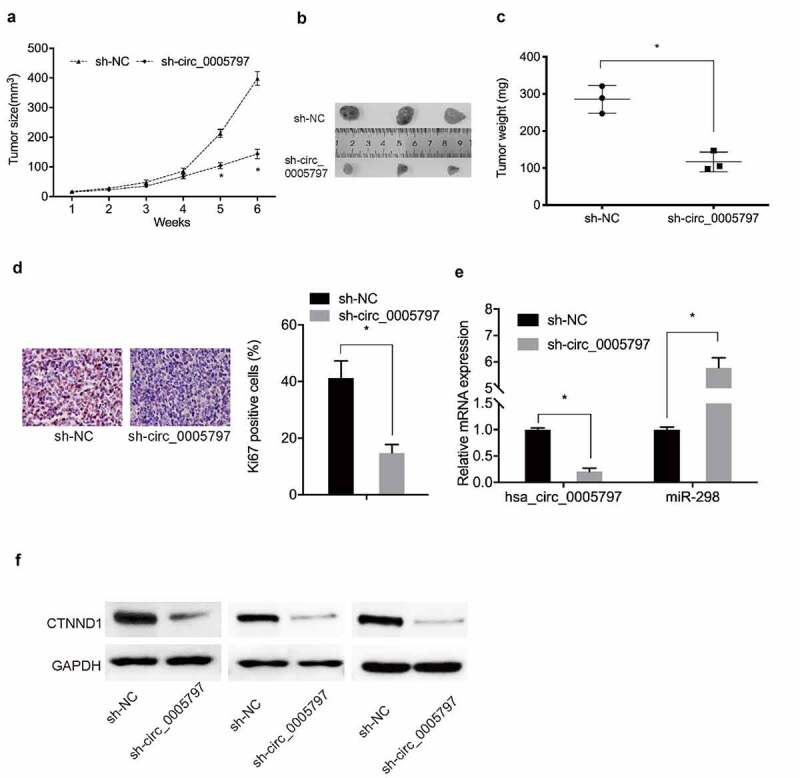
**Hsa_circ_0005797 suppressed tumor growth *in vivo***. (a-c) Mouse xenograft tumor volume and weight data. (d) Ki67 expression levels in xenograft tumors. (e, f) Hsa_circ_000579, miR-298, and CTNND1 expression levels in xenograft tumors. *P < 0.05.

To further characterize hsa_circ_0005797 during *in vivo*, a xenograft nude mouse model was generated. Compared with sh-NC group, hsa_circ_0005797 silencing significantly decreased *in vivo* tumor weight and volume (Figure 7a-7c). Immunohistochemistry (IHC) data showed that Ki67 levels were reduced in xenograft tumors from the sh-circ_0005797 group (Figure 7(d)). Moreover, sh-circ_0005797 injections decreased *in vivo* hsa_circ_0005797 and CTNND1, but increased miR-298 levels (Figure 7(e,f)). These observations suggested hsa_circ_0005797 facilitated EC tumor growth via miR-298/CTNND1 signaling.

## Discussion

In recent years, growing evidence indicated that circRNAs are key players in diverse biological processes and diseases, as well as in various types of cancer, including EC [19]. For example, circ_PUM1 promoted EC development by targeting miR-136/NOTCH3 signaling [20]. Similarly, hsa_circRNA_0001776 inhibited EC proliferation and promoted apoptosis by downregulating LRIG2 via miR-182 sponging [21]. Also, circTNFRSF21 promoted EC pathogenesis by modulating miR-1227-MAPK13/ATF2 signaling [22]. In our study, hsa_circ_0005797 was significantly elevated in EC tissues and cell lines. Elevated hsa_circ_0005797 expression was associated with advanced FIGO staging, and lymph node metastasis in patients with EC. Furthermore, hsa_circ_0005797 silencing effectively inhibited EC cells growth and invasion in vitro and reduced the malignancy of EC cells in vivo. Thus, our study suggested hsa_circ_0005797 is closely participated in EC progression, which might be a potential target for EC treatment.

Increasing studies showed that circRNAs play important functions during disease progression by directly binding to the 3’-UTR of target miRNAs and then inhibiting their transcription [23]. Hence, we explored whether hsa_circ_0005797 acted as a miRNA sponge. Through bioinformatics analysis, dual-luciferase reporter, and RIP assay et al, we proved that hsa_circ_0005797 served as a sponge of miR-298 in EC. Previous reports indicated miR-298 putatively functioned as a tumor repressor in several cancers. For example, miR-298 exhibited diagnostic and therapeutic potential in predicting doxorubicin chemoresistance in breast cancer [24]. Also, miR-298 inhibited epithelial ovarian cancer malignant phenotypes by regulating EZH2 levels [25]. Similarly, lncRNA GAS6-AS2 stimulated bladder cancer proliferation and metastasis via GAS6-AS2/miR-298/CDK9 signaling [26]. Herein, we showed miR-298 was reduced in EC, and its downregulation rescued the influence of hsa_circ_0005797 interference on EC cells proliferation and invasion abilities. Moreover, increased miR-298 expression suppressed EC cell proliferation and invasion in vitro. These findings uncovered new miR-298 functions in EC and extended the regulatory network of circRNA/miRNAs in this cancer, thereby contributing to disease understanding and etiology.

Next, we further investigate mechanisms underpinning hsa_circ_0005797/miR-298 signaling in EC. Through bioinformatics analysis, dual-luciferase reporter et al, CTNND1 was identified as a miR-298 target. Catenin delta-1 (CTNND1), as a member of the cadherin-catenin complex, directly binding to the cytoplasmic tail of E-cadherin via the former’s conserved juxtamembrane domain [27]. Recently, increasing studies showed that CTNND1 could function as an oncogene in cancers (including EC) by regulating various signaling pathways such as Wnt pathway et al [28]. For example, circ_0000043 promoted EC growth by regulating miR-1271-5p/CTNND1 signaling [29]. Also, hsa_circ_0002577 promoted EC by modulating miR-197/CTNND1 signaling and activating the Wnt/β-catenin pathway [30].

Here, we demonstrated CTNND1 functioned as a target of miR-298. CTNND1 overexpression attenuated the inhibitory effects of miR-298 mimics toward EC cells proliferation and invasion. Moreover, this overexpression of significantly attenuated the suppressive effects of hsa_circ_0005797 toward EC phenotypes. These observations indicated that hsa_circ_0005797 acted as an oncogenic circRNA promotes EC cells growth and invasion by regulating miR-298/CTNND1 signaling.

## Conclusion

Overall, we characterized the novel circRNA hsa_circ_0005797 as promoting EC growth and invasion by competitively binding to miR-298 via upregulated CTNND1 expression. These findings provide encouraging therapeutic targets for EC therapy.

## Data Availability

The data sets used and analyzed during the current study are available from the corresponding author on reasonable request.

## Highlights

1. hsa_circ_0005797 is highly expressed in EC
2. hsa_circ_0005797 promotes EC cells progression
3. hsa_circ_0005797/miR-298/CTNND1 axis in EC

## Abbreviations

circRNA; Circular RNA; EC: endometrial cancer; ncRNAs: non-coding RNAs; miRNA: microRNA; CTNND1: Catenin delta 1; siRNA: small interfering RNA; HEEC: human endometrial endothelial cells; DMEM: Dulbecco’s Modified Eagle Medium; FBS: fetal bovine serum; QRT-PCR: Quantitative real-time polymerase chain reaction; SDS-PAGE: sodium dodecyl sulfate-polyacrylamide electrophoresis; PVDF: polyvinylidene difluoride; CCK-8: Cell Counting Kit-8; WT: wild type, MUT: mutant type; 3’-UTR: 3’-Untranslated region; RIP: RNA immunoprecipitation; FIGO: Federation of Gynecology and Obstetrics; ceRNA: competitive endogenous RNAs.
